# Non-hormonal interventions for hot flushes in women with a history of breast cancer

**DOI:** 10.1590/S1516-31802013000100029

**Published:** 2013-04-01

**Authors:** Gabriel Rada, Daniel Capurro, Tomas Pantoja, Javiera Corbalán, Gladys Moreno, Luz María Letelier, Claudio Vera

## Abstract

**BACKGROUND:**

Hot flushes are common in women with a history of breast cancer. Hormonal therapies are known to reduce these symptoms but are not recommended in women with a history of breast cancer due to their potential adverse effects. The efficacy of non-hormonal therapies is still uncertain.

**OBJECTIVE:**

To assess the efficacy of non-hormonal therapies in reducing hot flushes in women with a history of breast cancer.

**METHODS:**

*Search methods:* We searched the Cochrane Breast Cancer Group Specialised Register, CENTRAL (The Cochrane Library), Medline, Embase, Lilacs, CINAHL, PsycINFO (August 2008) and WHO ICTRP Search Portal. We handsearched reference lists of reviews and included articles, reviewed conference proceedings and contacted experts.

*Selection criteria:* Randomized controlled trials (RCTs) comparing non-hormonal therapies with placebo or no therapy for reducing hot flushes in women with a history of breast cancer.

*Data collection and analysis:* Two authors independently selected potentially relevant studies, decided upon their inclusion and extracted data on participant characteristics, interventions, outcomes and the risk of bias of included studies.

**MAIN RESULTS:**

Sixteen RCTs met our inclusion criteria. We included six studies on selective serotonin (SSRI) and serotonin-norepinephrine (SNRI) reuptake inhibitors, two on clonidine, one on gabapentin, two each on relaxation therapy and homeopathy, and one each on vitamin E, magnetic devices and acupuncture. The risk of bias of most studies was rated as low or moderate. Data on continuous outcomes were presented inconsistently among studies, which precluded the possibility of pooling the results. Three pharmacological treatments (SSRIs and SNRIs, clonidine and gabapentin) reduced the number and severity of hot flushes. One study assessing vitamin E did not show any beneficial effect. One of two studies on relaxation therapy showed a significant benefit. None of the other non-pharmacological therapies had a significant benefit. Side-effects were inconsistently reported.

**AUTHORS’ CONCLUSIONS:**

Clonidine, SSRIs and SNRIs, gabapentin and relaxation therapy showed a mild to moderate effect on reducing hot flushes in women with a history of breast cancer.

**This is the abstract of a Cochrane Review published in the Cochrane Database of Systematic Reviews (CDSR) 2012, issue 11, art. No. CD004923. DOI:** 10.1002/14651858.CD004923.pub9 (http://onlinelibrary.wiley.com/doi/10.1002/14651858.CD004923.pub2/abstract?systemMessage=Wiley+Online+Library+will+be+disrupted+on+17+December+from+at+23%3A00+GMT+%2818%3A00+EST%29+for+essential+maintenance+for+approximately+one+hour). For full citation and authors details see reference 1

**The full text is freely available, for Latin America and the Caribbean, from:** http://cochrane.bvsalud.org/cochrane/show.php?db=reviews&mfn=2953&id=CD004923&lang=pt&dblang=&lib=COC&print=yes (this link may be temporary)
